# Electrophysiological and Alarm Responses of *Solenopsis invicta* Buren (Hymenoptera: Formicidae) to 2-Ethyl-3,5-dimethylpyrazine

**DOI:** 10.3390/insects10120451

**Published:** 2019-12-13

**Authors:** Ya-Ya Li, Deguang Liu, Li Chen

**Affiliations:** 1 College of Plant Protection, Northwest A & F University, Yangling, Xianyang 712100, China; liyaya0107@163.com; 2 State Key Laboratory of Integrated Management of Pest Insects and Rodents, Institute of Zoology, Chinese Academy of Sciences, Beijing 100101, China; 3 State Key Laboratory of Crop Stress Biology for Arid Areas (Northwest A & F University), Yangling, Xianyang 712100, China

**Keywords:** red imported fire ant, bioassay, antennal response, myrmecology, social behavior, alarm pheromone

## Abstract

2-Ethyl-3,5-dimethylpyrazine is an isomer of 2-ethyl-3,6-dimethylpyrazine, the alarm pheromone component of the red imported fire ant, *Solenopsis invicta* Buren. The pyrazine was synthesized and its alarm activity was investigated under laboratory conditions. It elicited significant electroantennogram (EAG) activities, and released characteristic alarm behaviors in fire ant workers. The EAG and alarm responses were both dose-dependent. Two doses of the pyrazine, 1 and 100 ng, were further subjected to bait discovery bioassays. Fire ant workers excited by the pyrazine were attracted to food baits, and their numbers increased over time. Ants displayed very similar response patterns to both low and high doses of the pyrazine. The pyrazine impregnated onto filter paper disc attracted significantly more fire ant workers than the hexane control for all observation time intervals at the low dose, and in the first 15 min period at the high dose. The pyrazine loaded onto food bait directly tended to attract more fire ant workers than the hexane control. These results support the potential use of 2-ethyl-3,5-dimethylpyrazine to enhance bait attractiveness for the control of *S. invicta* in invaded regions.

## 1. Introduction

Chemical communication is of great importance in social organizations, which can have large impacts on survival and maintenance of integrity of ant colonies. When facing potential dangers, ants release alarm pheromones to alert nestmates for colony defense [1]. In response to alarm pheromones, ants show characteristic behaviors, such as attacking and biting the odor source or moving rapidly away from the odor source [2,3]. Furthermore, alarm pheromones may act as attractants, thus recruiting more workers for intensified attacks on intruders. Alarm responses can vary among species [3]. Generally, ants of those species with large and dense colonies often run toward the pheromone source with mandibles opened [4].

Ants have evolved a wide variety of compounds in communicating threats [5]. Pyrazines are a major group of pheromonal compounds isolated from mandibular glands and venom reservoir in ants [6,7,8]. Pyrazines originating from mandibular glands typically trigger alarm responses, whereas those from poison glands often serve a trail following function [7].

Pyrazines seem to be widespread among the two subfamilies, Ponerinae and Myrmicinae, of the Formicidae. Few species in Dolichoderinae, Ecitoninae, Ectatomminae, and Formicinae produce pyrazines as alarm and trail pheromones [8,9]. Most ant pyrazines are tri-substituted derivatives. Mono-, di-, and tetra-substituted pyrazines are much less common. Numerous dimethylalkylpyrazines, which show 2,5-dimethyl-3-alkyl or 2,6-dimethyl-3-alkyl substitution patterns, have been detected from mandibular gland secretions of a variety of ant species [7,8].

**Figure 1:**
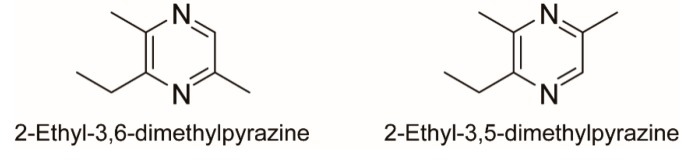
Chemical structures of pyrazines.

2-Ethyl-3,6-dimethylpyrazine (Figure 1) is a component of poison gland secretions of ant species in Myrmicinae (*Acromyrmex*, *Atta*, *Daceton*, *Manica*, *Messor*, *Metapone*, *Myrmica*, *Pheidole*, *Pogonomyrmex*, and *Tetramorium*) [8,10,11] and Ectatomminae (*Eciton burchelli*) [12], and primarily functions as a trail-following pheromone. There are several reports of 2-ethyl-3,6-dimethylpyrazine in mandibular glands of dolichoderine (*Iridomyrmex purpureus*), ectatommine (*Ectatomma* sp.) and ponerine (*Odontomachus bauri*, *Pachycondyla indica*, *Pachycondyla obscuricornis*, and *Pachycondyla striata*) ants [9,13,14]. No functions had been attributed to this compound in ants until Vander Meer et al. [15] reported its isolation and identification from the mandibular gland of the red imported fire ant, *Solenopsis invicta*. 2-Ethyl-3,6-dimethylpyrazine elicited rapid and erratic movement of fire ant workers, apparently functioning as an alarm pheromone.

2-Ethyl-3,5-dimethylpyrazine (Figure 1), an isomer of 2-ethyl-3,6-dimethylpyrazine, is a mandibular gland component of ponerine ants, *Odontomachus brunneus* [16], *O. chelifer* [8], *O. troglodytes* [17]. Unlike other pyrazine components from *O. troglodytes* workers, however, this pyrazine failed to release significant alarm behaviors [17]. Because 2-ethyl-3,6-dimethylpyrazine is commercially-available as a mixture with its isomer of 2-ethyl-3,5-dimethylpyrazine (ca. 60% 3,5-dimethyl isomer and 40% 3,6-dimethyl isomer), the synthetic mixture of 2-ethyl-3,6(or 5)-dimethylpyrazine has been used for most electrophysiological and behavioral studies [15,18,19,20,21,22,23]. Bioassays with isomers purified by preparative GC or HPLC demonstrated that 2-ethyl-3,6-dimethylpyrazine had a relatively lower active response threshold than 2-ethyl-3,5-dimethylpyrazine and the mixture of the two isomers [15,18].

A previous study has reported the electroantennogram (EAG) and behavioral responses of *S. invicta* workers to synthetic 2-ethyl-3,6-dimethylpyrazine [24]. However, very limited information on alarm activity of 2-ethyl-3,5-dimethylpyrazine is available for fire ants in the literature. In the present study, we aimed to examine both EAG and behavioral responses of fire ant workers to the synthetic pyrazine, and further test for attractiveness of the synthetic pyrazine-enhanced baits.

## 2. Materials and Methods

### 2.1. Ant Sources and Colony Maintenance

Mature *S. invicta* colonies were collected from the campus of South China Agricultural University (Guangzhou, China). Each colony with workers, broods, and queens was maintained in fluon-coated plastic trays through providing Milli-Q water and 10% honey solution *ad libitum*, and frozen crickets, *Gryllus testaceus* Walker every other day. Colonies were reared in a rearing room at 27 °C, 75% relative humidity, and a photoperiod of L:D 14:10 h.

### 2.2. Chemical Synthesis

2-Ethyl-3,5-dimethylpyrazine was synthesized as described in Fang and Cadwallader [25]. To a mixture of 2-chloro-3,5-dimethylpyrazine (5 g, 35.06 mmol) and ferric acetylacetonate (1.3 g, 3.68 mmol) in N-methyl-2-pyrrolidone (50 mL), EtMgBr (7.0 g, 52.51 mmol) was added dropwise at 0 °C under N_2_ atmosphere. The reaction mixture was stirred at 0 °C for 2 h. When the LC-MS analysis showed that the reaction was completed, the mixture was quenched with EtOAc and filtered. The organic phase was washed with saturated brine (20 mL × 5), dried over anhydrous Na_2_SO_4_, and evaporated. Flash chromatography of the residue with hexane/EtOAc = 30:1 as eluent yielded 2-ethyl-3,5-dimethylpyrazine (1.56 g, 11.47 mmol, 32.7% yield) as a colorless liquid. MS (EI), *m*/*z* (%) 136 (76, M^+^), 135 (100), 121 (6), 108 (13), 80 (3), 67 (5), 56 (19); ^1^H NMR: (400 MHz, CDCl_3_): *δ* 8.17 (s, 1H), 2.77 (q, *J* = 7.3 Hz, 2H), 2.50 (s, 3H), 2.45 (s, 3H), and 1.25 (t, *J* = 7.6 Hz, 3H) ppm.

### 2.3. Antennal Sensitivity Recordings

The antennal sensitivity of *S. invicta* workers to 2-ethyl-3,5-dimethylpyrazine was evaluated by conventional EAG methods as described in previous studies [18,24,26]. Briefly, an isolated ant head with antennae was connected between two glass electrodes filled with Ringer’s solution modified by Tween^®^ 80 [27]. The indifferent electrode was inserted into the open side of the head while the recording electrode was brought into contact with the antennal tip. Platinum wires were used to maintain the current between the antennal setting and a Syntech EAG Combi probe. The pre-amplified analog signal was converted into digital signal by a data acquisition controller IDAC-4, and analyzed with the software EAG 2000 (all from Syntech, Kirchzarten, Germany). A continuous air flow, which was purified through activated charcoal and humidified, was directed through a glass tube over the antennal preparation at 800 mL/min. An aliquot of 10 μL of solution of 2-ethyl-3,5-dimethylpyrazine in hexane was dropped on a filter paper strip (4 × 40 mm) placed in the shaft of a Pasteur pipette. Odor stimuli were then delivered by connecting the Pasteur pipette to a small hole in the wall of the glass tube as 0.2 s puff generated by an air stimulus controller CS-55 (Syntech, Kirchzarten, Germany). Fresh stimulus pipettes were prepared before each set of experiments.

Serial dilutions of 2-ethyl-3,5-dimethylpyrazine in hexane were prepared to make 0.01, 0.1, 1, 10, and 100 µg/µL solutions. The pyrazine solutions from low to high concentrations were applied to the same antennal preparation. A blank stimulus (solvent control) was presented before testing the compound. In addition to a solvent control, five doses (0.1, 1, 10, 100, or 1000 μg) were tested. EAG recordings were obtained from 8 antennal preparations for each dose.

### 2.4. Alarm Response

The alarm sensitivity of *S. invicta* workers to 2-ethyl-3,5-dimethylpyrazine was determined with the same method as described in previous reports [18,24]. We used the number of ants showing aggressive behaviors such as rapid, undirected running as a measure of alarm responses [18]. Briefly, 0.1 g of workers (approximately 100–150) were added to a plastic cup (9 cm tall × 14 cm i.d.) with the inside wall coated with Fluon to prevent escape. A 1-cm^3^ sugar-agar block (10% sugar water + 1% solidified agar) and a water tube were provided in the cup to allow ant feeding. A filter paper strip (1 × 3 cm) folded into a triangle was put in an empty space in the bottom of the cup. Further 10 × dilutions of 0.01 µg/µL solution of 2-ethyl-3,5-dimethylpyrazine were made to prepare 0.01, 0.1, and 1 ng/µL solutions. While ants were settled down in a quiescent group, 10 µL of sample solutions were gently loaded onto the filter paper strip. The number of workers that were running out of the group and/or displaying disoriented alarm behaviors within 2 min was recorded. Workers of a test cup were then given 30 min to recover prior to the next test. A new filter paper strip was used for each test. Alarm tests were conducted with the solvent control first and in order from low to high doses (0.1, 1, 10, 100, and 1000 ng). The experiment was replicated 10 times with ants from 10 different colonies.

### 2.5. Enhancement in Bait Attraction

The activity of 2-ethyl-3,5-dimethylpyrazine in enhancing bait attraction was evaluated using food block tests as described in previous reports [18,24]. In brief, 0.5 g of *S. invicta* workers were transferred into a plastic tray (55 cm diameter at the bottom) with a moisturized flat cotton wad in a Petri dish (d = 6 cm) at the center. Filter paper disks (4 cm diameter) were placed on three aluminum foil disks (5 cm diameter) that were 20 cm away from the center of the tray and at an equal distance from each other. Ants were allowed for acclimatization for 2 h. One block of hotdog (0.1 g) as a bait was then placed onto the filter paper disk. Immediately, 10-μL hexane was applied to both the filter paper disk and the hotdog block (5 μL each) on an aluminum disk serving as control. For the other two hotdog blocks, 10 μL of pyrazine solutions were loaded either onto the filter paper disk or onto the food block serving as treatments. The numbers of ants on a filter paper disk were counted 2, 5, 15, 30, and 45 min after sample loading. The experiment was replicated 8 times with ants from 8 different colonies. Two doses (i.e., 1 and 100 ng) of 2-ethyl-3,5-dimethylpyrazine were tested.

### 2.6. Statistical Analyses

The EAG amplitudes elicited by the pyrazine were corrected by subtracting EAG response to the solvent control. Similarly, the alarm behavior data obtained from pyrazine treatments were corrected by subtracting the number of ants responding to the solvent control. The corrected EAG data and alarm behavioral data were both analyzed for dose-response significance using the PROC REG procedure of the SAS statistical software [28]. Behavioral data sets were further analyzed with one-way repeated-measures ANOVA followed by the Tukey–Kramer HSD test (*p* < 0.05). Mean numbers of ants on different food baits at each observation time period were compared by using ANOVA followed by the Tukey–Kramer HSD test (*p* < 0.05) [28].

## 3. Results

### 3.1. Antennal Sensitivity

**Figure 2:**
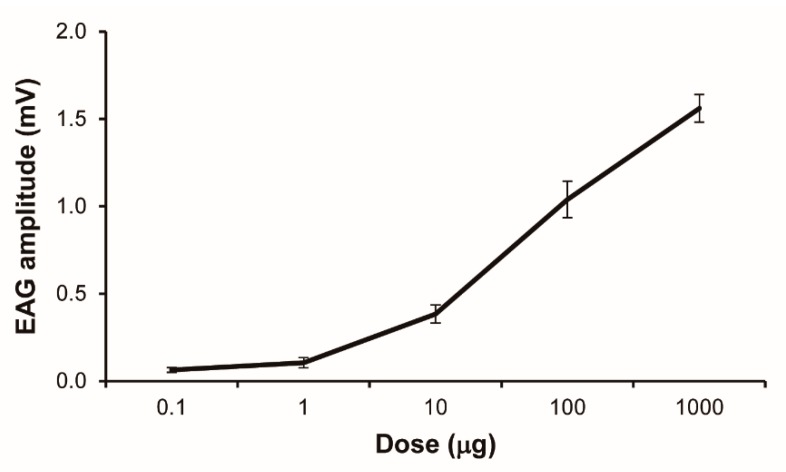
An electroantennogram (EAG) dose–response curve for *Solenopsis invicta* workers responding to 2-ethyl-3,5-dimethylpyrazine (mean ± SE, *n* = 8). The doses shown are the chemical mass that was impregnated on filter paper strips, not the actual doses delivered to the antennal preparation.

*Solenopsis invicta* workers showed a characteristic dose-dependent EAG response to 2-ethyl-3,5-dimethylpyrazine (Figure 2). There was a sharp increase in EAG response intensity when test doses of pyrazine increased from 10 to 1000 µg, but a saturated response was not reached. The corrected EAG data revealed a significant dose–response pattern (F_1,38_ = 199.96, *p* < 0.0001).

### 3.2. Alarm Activity

**Figure 3:**
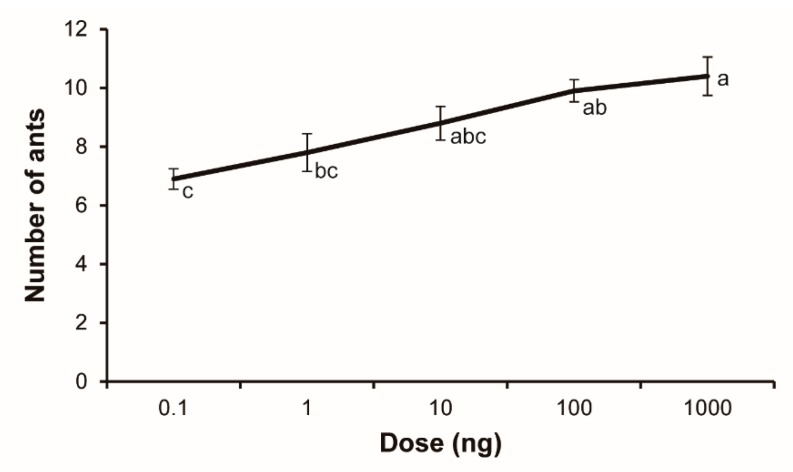
Alarm dose–response curve for *Solenopsis invicta* workers responding to 2-ethyl-3,5-dimethylpyrazine (mean ± SE, *n* = 10). The doses shown are the chemical mass that was impregnated on filter paper strips.

As shown in Figure 3, all doses of 2-ethyl-3,5-dimethylpyrazine elicited significant alarm behavioral responses of *S. invicta* workers. The alarm responses increased with increasing pyrazine doses. Statistical analysis of the corrected behavior data revealed a significant dose–response pattern (F_1,48_ = 30.50, *p* < 0.0001). The dose–response curve showed apparent saturation to the pyrazine at the highest dose tested.

### 3.3. Enhancement in Bait Attraction

**Figure 4:**
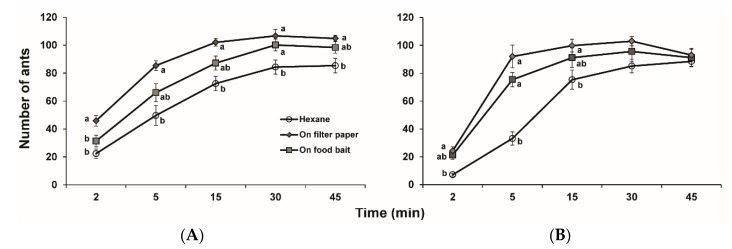
Attractiveness of 2-ethyl-3,5-dimethylpyrazine-treated hotdog baits to *Solenopsis invicta* workers. (**A**) 1 ng; (**B**) 100 ng. Means within the same time period with different letters are significantly different at *p* < 0.05, ANOVA followed by the Tukey-HSD test, and no letters indicate no differences between treatments.

Two doses of 2-ethyl-3,5-dimethylpyrazine were tested for possible enhancement in attraction of food baits to ants. The number of ants responding to food baits in control increased continuously over 45 min. At the dose of 1 ng, the number of ants in both pyrazine treatments increased in 30 min while that in control increased to a similar level in 45 min (Figure 4A). Significantly more ants were attracted to the bait with pyrazine on filter paper than the bait treated with pyrazine directly and control 2 min after application, and significantly more ants in both pyrazine treatments were attracted to the bait than control 30 min after application. For 5, 15, and 45 min observations, pyrazine applied onto filter paper and pyrazine directly applied to food bait attracted more ants than control. However, the difference in the number of ants was only statistically significant between the treatment with pyrazine applied onto filter paper and control. 

At the dose of 100 ng, the ants were quickly attracted to the pyrazine-treated food in 5 min, and reached a saturated response in 15 min (Figure 4B). Significantly more ants were attracted to both pyrazine-treated food baits than control 5 min after application. At 2 and 15 min observation time points, pyrazine applied onto filter paper and pyrazine directly applied to food bait attracted more ants than control, but the difference in number of ants was only statistically significant between the treatment with pyrazine applied to filter paper and control. There were no significant differences in number of ants between pyrazine treatments and control 30 min after application.

## 4. Discussion

Although perception of alarm pheromone has an important role in the chemical communication in ants, only few studies have tested EAG responses of ants to their alarm pheromones and related analogs [18,23,24,26,29,30]. EAG responses in fire ant workers under the treatment of 2-ethyl-3,5-dimethylpyrazine ranged from 0.06 to 1.56 mV (Figure 2). The dose-dependent EAG response pattern observed here was similar to that reported previously for 2-ethyl-3,6-dimethylpyrazine [24]. At the same dose, 2-ethyl-3,5-dimethylpyrazine generated a relatively lower EAG response intensity compared to 2-ethyl-3,6-dimethylpyrazine, indicating that olfactory neurons on the antennae of fire ants respond preferentially to its pheromone.

Alarm behavior is a basic feature of defensive strategy of ants, and may include a sequence of reactions to a source of danger [5,31]. Immediately after loading pyrazine to filter paper, ant workers were observed to run toward the odor sources with their mandibles wide open, showing typical “panic alarm” responses as previously described [2]. As the doses of pyrazine applied to filter paper strip increased, we observed more workers showing alarm reactions. Some alerted ants were observed biting the impregnated filter paper. These observations suggest that 2-ethyl-3,5-dimethylpyrazine can release the same alarm behaviors as the alarm pheromone component, 2-ethyl-3,6-dimethylpyrazine. The overall responses in fire ant workers to 2-ethyl-3,5-dimethylpyrazine appear to be dose-dependent (Figure 3). This response pattern was similar to that of 2-ethyl-3,6-dimethylpyrazine tested in a previous study [24].

The responses to alarm pheromone are concentration-dependent, as low concentrations generally release attraction of ants to the emission source [1]. Because fire ant workers contain only about 300 pg pheromonal pyrazine [15], two doses, 1 and 100 ng, were selected for food bait bioassays. Our results showed that both doses of 2-ethyl-3,5-dimethylpyrazine excited fire ant workers and attracted them to the vicinity of the food bait. Several minutes later, a large number of workers ran onto the food bait and started feeding. In low dose treatments, the pyrazine loaded onto filter paper disk attracted significantly more workers than the sample loaded onto the bait directly at first 2 min, possibly due to faster evaporation of pyrazine from the filter paper. The pyrazine kept workers in an agitation status while feeding on the food bait. The number of workers feeding on the food bait in control increased continuously and stabilized after 30 min. The number of workers responding to high doses of pyrazine quickly increased to a high level at first 5 min. Because there were no differences at 30 and 45 min between high-dose treatments and control, the workers excited by pyrazine might recruit more nestmates to control food baits as well. The pattern for the increase in the number of workers drawn to the food bait by 2-ethyl-3,5-dimethylpyrazine over time is in accord with that for 2-ethyl-3,6-dimethylpyrazine and other pyrazine analogs [18,24].

Previous field studies have demonstrated that traps baited with pheromonal pyrazines can increase species-specific detection of the little fire ant, *Wasmannia auropunctata* [32]. Both laboratory and field studies indicated that the addition of (Z)-9-hexadecenal, a trail pheromone component of Argentine ant, *Linepithema humile* (Mayr), could enhance food consumption [33,34]. The alarm pheromone has proved to not only improve the attractiveness and harvest of the pheromone-enhanced bait, but also to stimulate a general increase in ant activities, resulting in a rapid discovery of unenhanced bait placed nearby [35,36]. These pyrazines may be able to mask the odor of insecticidal baits to increase feeding. Peanut butter is rich in sugars, lipids, and proteins, and releases a number of volatile alkylpyrazines, including 2-ethyl-3,5-dimethylpyrazine and 2-ethyl-3,6-dimethylpyrazine [37], which makes it a preferable food source for preparing baits. These two pyrazine isomers can be incorporated in peanut butter for potential control applications. Pyrazine-modified peanut butter could be more species-specific and longer lasting for detection of fire ant workers. The potential use of 2-ethyl-3,5(or 6)-dimethylpyrazine-incorporated peanut butter in detection and control of *S. invicta* in the invaded regions awaits further field investigations.

## 5. Conclusions

Our study showed that synthetic 2-ethyl-3,5-dimethylpyrazine elicited dose-dependent EAG and alarm behavioral responses. These response patterns were similar to those of the alarm pheromone component of *S. invicta* workers, 2-ethyl-3,6-dimethylpyrazine. Impregnation of 2-ethyl-3,5-dimethylpyrazine to food bait resulted in higher number of fire ant workers being attracted to the treated food bait. Our data suggest that this alarm pheromone isomer has the potential to be incorporated as a bait enhancer for management of *S. invicta* in introduced ranges.
